# Device measured physical activity before pregnancy and the risk of adverse pregnancy outcomes in the HUNT study: a population-based cohort study

**DOI:** 10.1186/s12884-025-07779-7

**Published:** 2025-06-07

**Authors:** Laura Vatn Slapgaard, Signe Nilssen Stafne, Vegar Rangul, Ingrid Aanesland Dahle, Julie Horn

**Affiliations:** 1 HUNT Research Centre, Department of Public Health and Nursing, Norwegian University of Science and Technology, Trondheim, Norway; 2 Department of Clinical and Molecular Medicine, Norwegian University of Science and Technology, Trondheim, Norway; 3 Clinic of Rehabilitation, St. Olavs University Hospital, Trondheim, Norway; 4 Levanger Hospital, Nord-Trøndelag Hospital Trust, Levanger, Norway; 5 Department of Obstetrics and Gynecology, Levanger Hospital, Nord-Trøndelag Hospital Trust, Levanger, Norway

**Keywords:** Accelerometer, Gestational diabetes, Hypertensive disorders of pregnancy, Physical activity, Pregnancy complications, Preterm birth, Small-for-gestational age

## Abstract

**Background:**

Adverse pregnancy outcomes (APOs), including gestational diabetes mellitus (GDM), hypertensive disorders of pregnancy (HDP), small for gestational age (SGA) offspring, preterm birth and placental abruption, pose significant short and long-term health consequences for mothers and their offspring. Engaging in physical activity may reduce the risk of APOs. We aimed to examine the association between objectively measured physical activity before pregnancy and risk of APOs.

**Methods:**

The study population consisted of women with one or more singleton births registered in the Medical Birth Registry of Norway (MBRN) after participating in the fourth survey of the Trøndelag Health Study (HUNT4, 2017–2019). HUNT4 provided data on accelerometer measures on physical activity. We used multivariable adjusted logistic regression models to estimate associations between physical activity (total daily physical activity and metabolic equivalent of task (MET) min/week of moderate to vigorous physical activity (MVPA)) and risk of APOs (GDM, HDP, SGA offspring, preterm birth and/or placental abruption).

**Results:**

Among 700 women included in the study population, 145 (20.7%) experienced at least one APO. Compared to women in the lowest tertile of total physical activity/day, those in the highest tertile had lower odds of GDM (OR 0.19; 95% CI: 0.38–0.93) and potentially lower odds of any APO (OR 0.69; 95% CI: 0.43–1.11). Women with higher levels of total daily physical activity and higher levels of MVPA had lower odds for the composite outcome HDP, GDM, and/or SGA (OR 0.49; 95% CI: 0.28–0.87, highest tertile compared to lowest tertile and OR 0.53; 95% CI: 0.28–1.01, > 1000 MET minutes/week compared to < 500 MET minutes/week).

**Conclusions:**

Increased prepregnancy physical activity, including total daily activity, may reduce the risk APOs. Promoting preconceptional everyday activity could be key to improving pregnancy health. Larger studies are needed to confirm these findings.

**Supplementary Information:**

The online version contains supplementary material available at 10.1186/s12884-025-07779-7.

## Background

Adverse pregnancy outcomes (APOs), including hypertensive disorders of pregnancy (HDP), gestational diabetes mellitus (GDM), small for gestational age (SGA) offspring, placental abruption and preterm birth, are major contributors to maternal and offspring morbidity and mortality during pregnancy, delivery and the postpartum period. Furthermore, APOs have long-term implication on the health of mothers and their offspring. Women experiencing APOs have an elevated risk of developing cardiometabolic disease later in life [1–3] and exposure to APOs in utero is associated with long-term adverse effects on offspring cardiometabolic health [4–6]. Understanding the factors that contribute to these complications is essential for developing effective preventive strategies, improving maternal and child health outcomes, and reducing the societal burden associated with APOs.

Physical activity is a crucial component of a healthy lifestyle, encompassing any movement produced by skeletal muscles that expends energy [7]. The World Health Organization (WHO) has established guidelines, stating that all adults should engage in minimum 150–300 min of moderate-intensity physical activity, 75–150 min of vigorous-intensity physical activity, or some equivalent combination of moderate-intensity and vigorous-intensity aerobic physical activity per week [7]. Only one in four adults meet these recommendations for aerobic physical activity [8]. However, even light-intensity physical activity, such as slow walking, may provide significant improvements in overall health and contribute to increased longevity [9, 10].

Physical activity has consistently been linked to improved vascular health due to its ability to induce shear stress on blood vessels. Shear stress, the force exerted by blood flow on the vascular walls, stimulates the production of nitic oxide and other vasodilatory substances, which is shown to improve arterial elasticity and promote angiogenesis. These molecular changes are not only beneficial to prevent cardiovascular diseases later in life [11], but also in pregnancy, where vascular adaptions in placental and systemic circulation are crucial for proper remodeling of uteroplacental arteries. The remodeling is essential to ensure adequate blood flow to the placenta and meet the heightened circulatory demand of the growing fetus [12].

Several previous studies have examined the impact of physical activity before and during pregnancy on risk of APOs and indicate an association between increased levels of physical activity and reduced risk of preeclampsia, GDM and preterm birth [13–17]. Higher levels of prepregnancy physical activity were linked to a greater risk reduction than higher levels of physical activity during pregnancy [14, 16].

However, most research exploring the association between pre-pregnancy physical activity and APOs relies on self-reported physical activity, which may be prone to misclassification. Furthermore, only a few studies have included data on light intensity physical activity, such as walking. Better understanding of the potential benefits of light physical activity in preventing APOs is important, as light physical activity may be easier to incorporate into daily life compared to recommendations for moderate to vigorous physical activity.

The aim of this study is to investigate the association of device measured physical activity before pregnancy with the risk of APOs in women participating in the fourth survey of the population-based Trøndelag Health study (HUNT4).

## Methods

### Study design and population

The study population consists of women who have participated in HUNT4 (2017–2019) and who have been registered with one or more singleton births in the Medical Birth Registry of Norway (MBRN) between their participation in HUNT4 and 31.12.2022. Linkage of the two data sources was possible using the unique 11-digit personal identity number of Norwegian residents.

HUNT4 is a large population-based cohort study in the northern part of Trøndelag county, Norway. All residents aged 20 years or older, were invited to participate. The study included more than 30,000 women. Data were collected using questionnaires regarding lifestyle and health-related factors, clinical examinations and physical activity assessment using accelerometer recording [18]. Participation rate in the HUNT4 varied according to age and sex and was between 43.5 and 59.1% for women younger than 50 years [18].

The MBRN is a mandatory national health registry, containing data of all births in Norway since 1967, including information on the pregnancy complications of interest in this study [19].

**Fig. 1:**
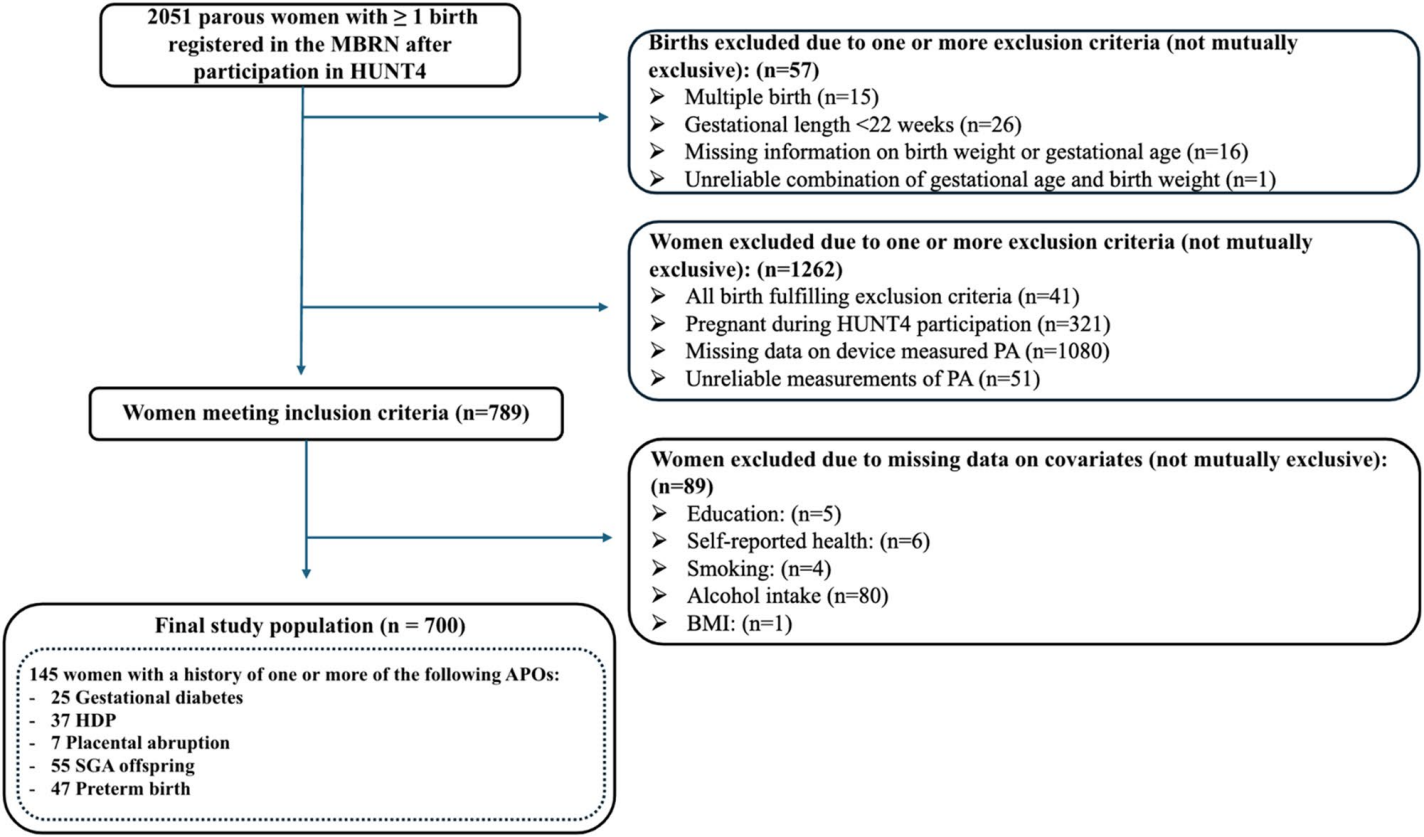
Study population. BMI, Body mass index; HDP, hypertensive disorders of pregnancy; HUNT4, Trøndelag Health Study; MBRN, Medical Birth Registry of Norway; PA, physical activity; SGA, small for gestational age

A total of 2051 women had at least one birth registered in the MBRN since their participation in HUNT4. We excluded births due to one or more of the following non-mutually exclusive criteria: multiple birth, gestational length < 22 weeks, missing information on birth weight or gestational age or an unreliable combination of gestational age and birth weight. This resulted in the exclusion of 41 women for whom all of their births fulfilled the exclusion criteria. We furthermore excluded participants for the following non-mutually exclusive criteria: pregnant during HUNT4 participation (*n* = 321), missing data on device measured PA (*n* = 1080), unreliable measurements of PA (*n* = 51), and missing data on covariates (*n* = 89), which left 700 women in the final study population (Fig. 1). Missing data on PA were primarily due to logistical constraints, such as delays in sensor returns and losses during the return mailing process. The median time between HUNT4 participation and the first subsequent birth was 23 months (interquartile range 12–38 months).

### Physical activity assessment

HUNT4 included detailed measures of physical activity. This was conducted with participants wearing two tri-axial AX3 accelerometers (Axivity, Newcastle, UK). The accelerometers were placed on the right thigh and on the lower back, and participants were encouraged to wear them continuously, 24 h a day, for seven consecutive days. Only days with complete 24-hour accelerometer recordings were included in the analysis. Data from two accelerometers were combined and segmented into 5 s windows. A validated eXtreme Gradient Boosting (XGBoost) machine learning model was used to classify physical activity behaviors, including lying down, sitting, standing, walking in three different paces (slow walking: < 4 km/h, moderate walking: 4,1–5,4 km/h, fast walking: > 5,5 hm/h), running and cycling. The model has been validated with free-living conditions showing an overall high accuracy [20, 21]. Within the study population, participants wore the accelerometers for an average of 5.6 days. Participants with at least three valid days of accelerometer recordings were included in this study.

For this study, physical activity data were analyzed and presented as total daily physical activity (minutes/day) and weekly moderate to vigorous physical activity (MVPA). Total daily physical activity (min per day) was categorized by adding time spent walking (including slow walking, which falls within the light-intensity range), running and cycling.

MVPA was measured in metabolic equivalents of task (MET) minutes/week. To find the MET minutes/week, each activity was given a MET-rating according to intensity. A rating of 2.8 was assigned for slow walking, 3.5 for moderate walking, 4.5 for fast walking, 6.5 for running and 5.8 for cycling [22]. We then divided the summarized values by the number of days the accelerometer was used and then multiplied by 7. MVPA was defined according to common physical activity recommendations based on activities exceeding 3 metabolic equivalents of task (METs), including moderate walking (3.5 METs), fast walking (4.5 METs), running (6.5 METs), cycling (5.8 METs).

We categorized total daily activity into percentiles: below the 33rd percentile (88 min activity/day or less), between the 33rd and 66th percentiles (between 88 min and 115 min of activity/day), and above the 66th percentile (above 115 min of activity/day). Similarly, MVPA was divided into three groups based on weekly MET-minutes: less than 500 MET-min/week, 500–1000 MET-min/week, and more than 1000 MET-min/week. The basis for this categorization of MVPA is that 500–1000 MET minutes per week is an internationally recognized minimum recommendation for weekly physical activity [23].

To compare HUNT4 participants with and without available accelerometer measurements, we also assessed self-reported physical activity levels, categorized as active or inactive. Responses to questions regarding the frequency, intensity, and duration of physical activity were combined to define inactivity as less than 150 min of physical activity per week.

### Outcome assessment

A history of APOs was retrieved from the MBRN and included a history of GDM, HDP (preeclampsia or gestational hypertension), placental abruption, SGA and/or preterm birth, in one or more pregnancies. Each woman contributed once to the analysis.

GDM is defined as abnormal glucose tolerance with onset, or first recognition, during pregnancy [24]. The Norwegian diagnostical criteria for GDM is fasting plasma glucose between 5.3 and 6.9 mmol/L and/or a two-hour plasma glucose level of 9.0–11.0 mmol/L after a 75 g oral glucose tolerance test [25]. We focused on HDP among women with new-onset hypertension in pregnancy (gestational hypertension and preeclampsia). Gestational hypertension is defined as new-onset of blood pressure ≥ 140/90 mm Hg after 20 weeks of gestation, while a diagnosis of preeclampsia additionally requires proteinuria or evidence of end-organ dysfunction [26]. Offspring with birth weight below the 10th percentile for gestational age and sex, based on Norwegian standards [27], were defined as SGA. Gestational age was determined via ultrasound dating or the last menstrual period, with second-trimester ultrasound screening conducted in nearly all pregnancies [28]. Preterm birth was defined as birth before 37 weeks of gestation.

### Covariate assessment

The following variables, related to lifestyle behaviors and the risk of APOs, were considered as potential confounders: age at participation in HUNT4, level of education, self-reported general health, cohabitation status (living with a partner or not), parity, smoking status, alcohol intake, and body mass index (BMI). BMI was calculated as weight in kilograms divided by the squared value of height in meters, based on standardized measurements of height and weight. We gathered data on parity from the MBRN, while the other covariates were obtained from HUNT4.

### Statistical analysis

The statistical analysis was performed using STATA/MP 18.0. Descriptive characteristics of the study population are presented as means and standard deviations (SD) for continuous variables and as numbers and percentages for categorical variables.

We estimated the association of level of physical activity before pregnancy and incidence of APO using logistic regression analysis. Model 1 was adjusted for age, Model 2 was adjusted for age, cohabitation status, education and parity, Model 3 was additionally adjusted for smoking, alcohol intake, BMI and self-reported general health. The results are presented as odds ratios (OR) and 95% confidence intervals (CI).

### Sensitivity analysis

We performed sensitivity analyses among women without self-reported moderate or severe impairment due to physical illness or mental health in questionnaires. In another sensitivity analysis, we included women with unreliable measurements of physical activity (< 3 min of walking ≥ 1 day), to evaluate the effect of excluding these women on the results. We also performed descriptive statistics of excluded and included population, to highlight any potential differences.

### Ethical approvals

All participants in HUNT4 have given informed written consent for the use of their data, including cross-linking with national registries. The Regional Committee for Medical and Health Research Ethics (REC Central) approved this study on December 7th, 2023 (reference no. 698119).

## Results

In our sample, there were 700 women with 824 births, of whom 145 women (20.7%) experienced one or more APO following their participation in HUNT4. A total of 122 women experienced one APO (17,4%), while 23 experienced two or more APOs (3.3%). Among the women with APOs, 37 (5.3%) experienced HDP, 25 (3.6%) experienced GDM, 7 women (1%) had a placental abruption, 55 (7.9%) gave birth to a SGA offspring, and 47 (6.7%) experienced a preterm birth. Table 1 presents participants’ physical activity metrics, including MVPA and total physical activity, according to baseline characteristics and history of APOs following HUNT4.

**Table 1:** Prepregnancy physical activity metrics at HUNT4 (2017–2019) participation according to baseline characteristics and later adverse pregnancy outcomes, *n* = 700

Characteristics	*N* (%)	MVPA (MET minutes/week)*	Total activity/day (min/day)*
**Age, years (mean = 28.3, SD = 4.4)**			
18–25	178 (25.4)	1044.1 (615.6)	97.9 (31.7)
> 25–30	303 (43.3)	1099.4 (643.5)	104.4 (30.7)
> 30–35	154 (22.0)	1094.7 (596.6)	110.9 (32.7)
> 35	65 (9.3)	1011.7 (560.2)	104.9 (28.0)
**Cohabitation status**			
Living with a partner	547 (78.1)	1037.4 (592.3)	105.0 (31.1)
Living without a partner	153 (21.9)	1214.9 (688.5)	101.2 (32.6)
**Education**			
Professional certificate, high school or less	219 (31.3)	974.4 (542.3)	100.1 (31.6)
Higher education	481 (68.7)	1122.5 (645.4)	106.1 (31.2)
**Parity**			
Nulliparous	359 (51.3)	1188.0 (648.1)	101.0 (32.4)
Parous	341 (48.7)	958.4 (563.0)	107.6 (30.1)
**Self-reported general health**			
Poor/fair	71 (10.1)	930.3 (526.6)	95.2 (27.6)
Good/excellent	629 (89.9)	1092.6 (626.2)	105.2 (31.7)
**Smoking status**			
Never smoked	451 (64.4)	1106.4 (659.8)	104.9 (33.0)
Ever smoker	249 (35.6)	1021.5 (532.3)	103.0 (28.5)
**Alcohol intake**			
No alcohol consumption	165 (23.6)	990.0 (639.9)	102.7 (31.7)
Any alcohol consumption	535 (76.4)	1102.8 (610.8)	104.7 (31.4)
**BMI, kg/m** ^**2**^			
Underweight/normal weight (< 24.9)	409 (58.4)	1153.7 (636.4)	107.4 (31.0)
Overweight (25-29.9)	181 (25.9)	1057.3 (622.2)	104.5 (32.7)
Obese (≥ 30)	110 (15.7)	819.0 (455.4)	91.8 (27.8)
**History of adverse pregnancy outcomes (APO) after HUNT4 participation**			
Women without APO	555 (79.3)	1080.5 (621.7)	105.7 (31.3)
Women with any APO	145 (20.7)	1059.5 (607.7)	98.3 (31.5)
HDP	37 (5.3)	1005.4 (674.6)	94.4 (35.6)
GDM	25 (3.6)	808.7 (380.8)	89.7 (22.0)
SGA	55 (7.9)	1106.2 (588.5)	98.2 (31.1)
HDP, GDM and/or SGA	103 (14.7)	979.2 (588.9)	93.1 (31.1)
Placental abruption	7 (1.0)	890.3 (617.2)	72.9 (20.9)
Preterm birth	47 (6.7)	1252.1 (604.5)	109.1 (28.1)

Values are numbers (percentages) unless otherwise noted

APO, adverse pregnancy outcome; BMI, body mass index; GDM, gestational diabetes mellitus; HDP, hypertensive disorders of pregnancy (preeclampsia and gestational hypertension); MET, metabolic equivalent of task; MVPA, moderate-vigorous physical activity; SD, standard deviation; SGA, small for gestational age offspring

*Total activity/day: Composed by slow, moderate and fast walking, running- and cycling/day

*MVPA, Moderate to Vigorous Physical activity (MET Total score): Composed by MET cycling, MET running, MET walking

At the time of HUNT4 participation, participants’ mean age was 28.3 years (SD 4.4 years). Roughly half of the participants were nulliparous, two thirds reported at least some college or university education, and 78.4% were living with a partner. General health (prepregnancy) was self-reported as poor/fair by 10.1% of the participants. The prevalence of overweight (BMI 25–29.9 kg/m2) and obesity (BMI ≥ 30 kg/m2) was 26.4% and 16.3%, respectively.

Physical activity levels varied according to socioeconomic, lifestyle, and health-related factors. Level of MVPA was highest in the age between > 25–30 years (1099.4 MET minutes/week) and lowest among women older than 35 years (1011.7 MET minutes/week). Total daily activity peaked in the age > 30–35 (110.9 min/day) and was lowest in the age between 18 and 25 years (97.9 min/day).

Women living with a partner tended to have lower levels of MVPA than those without. Higher education was generally linked to greater MVPA and total daily activity, while nulliparous women showed higher MVPA but lower total activity. Women reporting good/excellent health and never-smokers appeared to be more active across all metrics than those with poor/fair health or ever-smokers. Underweight/normal-weight women appeared to be the most active, while obese women had the lowest activity levels across all metrics.

Participants with uncomplicated pregnancies following HUNT4 participation tended to have higher MVPA and higher total activity/day than those experiencing any APO. However, women experiencing preterm birth showed the highest prepregnancy activity levels of all women across all metrics, especially for MVPA.

### Association between prepregnancy level of physical activity and risk of adverse pregnancy outcomes

The associations between prepregnancy physical activity measures and risk of APOs are presented in Table 2. Women in the highest tertile of total activity/day (> 66th percentile; >115 min/week) had 40% lower odds of any APO compared to those in the lowest tertile (< 33rd percentile) (OR 0.60; 95% CI: 0.38–0.95) in the age-adjusted model. However, this association was attenuated in the fully adjusted model (OR 0.69; 95% CI: 0.43–1.11).

**Table 2:** Association of prepregnancy physical activity with the risk of adverse pregnancy outcomes among HUNT4 participants (*n* = 700)

	Number of cases	Model 1^a^		Model 2^b^		Model 3^c^	
		OR	95% CI	OR	95% CI	OR	95% CI
**Any adverse pregnancy outcome**	**145**						
Total physical activity/day							
< 33 percentile	61	Ref.		Ref.		Ref.	
33–66 percentile	44	0.67	(0.44–1.05)	0.71	(0.45–1.10)	0.74	(0.47–1.16)
> 66 percentile	40	0.60	(0.38–0.95)	0.65	(0.41–1.03)	0.69	(0.43–1.11)
**MVPA (MET min/week)**							
< 500	25	Ref.		Ref.		Ref.	
500–1000	52	0.62	(0.36–1.07)	0.56	(0.32–0.98)	0.58	(0.33–1.03)
> 1000	68	0.73	(0.43–1.24)	0.61	(0.35–1.06)	0.67	(0.38–1.19)
**HDP**	**37**						
Total physical activity/day							
< 33 percentile	18	Ref.		Ref.		Ref.	
33–66 percentile	10	0.55	(0.25–1.22)	0.57	(0.25–1.26)	0.60	(0.27–1.37)
> 66 percentile	9	0.49	(0.21–1.13)	0.54	(0.23–1.25)	0.59	(0.25–1.41)
MVPA (MET min/week)							
< 500	8	Ref.		Ref.		Ref.	
500–1000	13	0.52	(0.21–1.29)	0.46	(0.18–1.16)	0.49	(0.19–1.27)
> 1000	16	0.57	(0.23–1.37)	0.46	(0.18–1.14)	0.52	(0.20–1.33)
**GDM**	**25**						
Total physical activity/day							
< 33 percentile	10	Ref.		Ref.		Ref.	
33–66 percentile	13	1.29	(0.55–3.02)	1.32	(0.55–3.16)	1.67	(0.66–4.21)
> 66 percentile	2	0.17	(0.04–0.78)	0.17	(0.04–0.79)	0.19	(0.38–0.93)
MVPA (MET min/week)							
< 500	5	Ref.		Ref.		Ref.	
500–1000	13	0.85	(0.29–2.47)	0.94	(0.32–2.78)	1.12	(0.37–3.44)
> 1000	7	0.36	(0.11–1.17)	0.41	(0.12–1.39)	0.55	(0.16–1.94)
**SGA**	**55**						
Total physical activity/day							
< 33 percentile	23	Ref.		Ref.		Ref.	
33–66 percentile	15	0.66	(0.34–1.31)	0.68	(0.34–1.35)	0.62	(0.31–1.26)
> 66 percentile	17	0.80	(0.41–1.55)	0.86	(0.44–1.67)	0.75	(0.38–1.48)
MVPA (MET min/week)							
< 500	8	Ref.		Ref.		Ref.	
500–1000	20	0.81	(0.34–1.91)	0.72	(0.30–1.72)	0.65	(0.27–1.58)
> 1000	27	0.99	(0.43–2.26)	0.81	(0.35–1.91)	0.73	(0.30–1.76)
**Preterm Birth**	**47**						
Total physical activity/day							
< 33 percentile	13	Ref.		Ref.		Ref.	
33–66 percentile	15	1.19	(0.55–2.56)	1.26	(0.58–2.73)	1.18	(0.54–2.58)
> 66 percentile	19	1.51	(0.72–3.16)	1.70	(0.81–3.58)	1.69	(0.78–3.63)
**MVPA (MET min/week)**							
< 500	6	Ref.		Ref.		Ref.	
500–1000	11	0.59	(0.21–1.64)	0.55	(0.20–1.55)	0.52	(0.18–1.49)
> 1000	30	1.48	(0.60–3.69)	1.34	(0.53–3.40)	1.29	(0.49–3.36)
**HDP, GDM and/or SGA**	**103**						
Total physical activity/day							
< 33 percentile	50	Ref.		Ref.		Ref.	
33–66 percentile	30	0.56	(0.34–0.93)	0.58	(0.35–0.97)	0.63	(0.38–1.05)
> 66 percentile	23	0.43	(0.25–0.73)	0.46	(0.27–0.80)	0.49	(0.28–0.87)
MVPA (MET min/week)							
< 500	20	Ref.		Ref.		Ref.	
500–1000	42	0.62	(0.34–1.13)	0.57	(0.31–1.05)	0.61	(0.33–1.15)
> 1000	41	0.55	(0.30-1.00)	0.46	(0.25–0.86)	0.53	(0.28–1.01)

APO, adverse pregnancy outcome; BMI, body mass index; CI, confidence interval; GDM, gestational diabetes mellitus; HDP, hypertensive disorder of pregnancy; MET, metabolic equivalent of task; MVPA, moderate-vigorous physical activity; OR, odds ratio; SGA, small for gestational age

Total physical activity/day percentiles: <33 = 0–89 min, 33–66 = 89–115 min, > 66 = > 115 min

a: adjusted for age. b: adjusted for age, cohabitation status, education and parity

c: additionally adjusted for smoking, alcohol intake, BMI and self-reported general health

For women in the highest tertile of total physical activity/day the odds of GDM were reduced by 81% compared to women in the lowest tertile (OR 0.19; 95% CI: 0.38–0.93) in the fully adjusted model.

We found no associations between prepregnancy physical activity and HDP, SGA or preterm birth, although the estimates suggested a lower odds of HDP and a higher odds of preterm birth among women in the highest tertial of total activity/day.

Since descriptive analyses indicated high levels of physical activity among women who later experienced preterm birth, but not among women with other APOs, we examined the composite outcome of HDP, GDM, and/or SGA in an additional analysis. Women in the highest tertile of total physical activity/day had 51% lower odds (OR 0.49; 95% CI: 0.28–0.87) of these APOs compared to those in the lowest tertile in the fully adjusted model. We found no clear associations between levels of MVPA and risk of APOs. However, compared to women with less than 500 MET minutes/week, women with more than 1000 MET minutes/week of MVPA appeared to have lower odds of experiencing the composite outcome HDP, GDM, and/or SGA (OR 0.53; 95% CI: 0.28–1.01).

### Sensitivity analysis

Restricting our analyses to women without self-reported moderate or severe impairment due to physical or mental health problems (*n* = 663) slightly strengthened most of the estimates compared to those in the main analysis (Supplemental Table 1). Sensitivity analyses including women with unreliable measurements of physical activity yielded almost unchanged estimates (Supplemental Table 2).

### Comparison of excluded and included HUNT4 participants

Supplemental Table 3 presents descriptive characteristics of excluded and included participants. A total of 1351 (65.9%) women were excluded from the study population, mainly due to missing data on device measured physical activity (*n* = 1080).

Women included in the study population were less likely to live with a partner, more likely to have higher education, and more often nulliparous compared to excluded women. They also had a higher proportion of never-smokers, reported any alcohol consumption more frequently, and had a lower proportion of overweight or obese individuals. Furthermore, a larger proportion of included women self-reported high physical activity (> 3 times/week, 22.7% vs. 17.2%). Among the included population, 52.4% were categorized as active (≥ 150 min of physical activity per week), compared to 46.3% of the excluded population.

## Discussion

The results from this prospective population-based cohort study with data on device measured physical activity before pregnancy showed an association between higher levels of daily physical activity and lower risk of the composite outcome GDM, HDP and/or SGA, which was determined post hoc. Higher daily physical activity particularly reduced the risk of GDM.

Our findings align with results of two previous meta-analyses that reported an inverse association between self-reported prepregnancy physical activity and the risk of GDM [15, 16]. A protective effect of physical activity before pregnancy on the risk of GDM is plausible, given that physical exercise has been consistently linked with improved insulin sensitivity [29]. Physical activity is widely recognized for its ability to enhance vascular function and lower blood pressure [11], effects that could contribute to reduced risk of APOs associated with placental dysfunction such as HDP and fetal growth restriction.

Studies that evaluated the association of prepregnancy physical activity and risk of HDP had mixed results. A previous study including women participating in the first survey of the HUNT study (HUNT1, 1984–1986) reported a tendency for reduced risk of preeclampsia for women physically active for 120 min/week or more (OR 0.6; 95% CI 0.3–1.2) [30], which is comparable to our findings on the association between physical activity and risk for HDP. A meta-analysis, comparing any physical activity vs. no physical activity, indicated a reduced risk of preeclampsia in case-control, but not in the included cohort studies [31]. Another systematic review and meta-analysis done by Aune et al. on the association between physical activity and risk of preeclampsia found a 35% reduced risk of preeclampsia in women with high versus low level of physical activity [14]. The Nurses’ Health Study II, a large cohort study from the USA, looked at the association between physical activity before pregnancy and the risk of HDP [13]. The study included 18,283 women, with 842 cases of preeclampsia and 905 cases of gestational hypertension, and demonstrated a 25% lower risk of HDP for women in the highest quartile compared with women in the lowest quartile of physical activity. However, this risk reduction appeared to be mainly driven by a reduced risk of gestational hypertension (RR 0.61, 95% CI 0.50-0,76), while there was no indication of an association between prepregnancy physical activity and the risk of preeclampsia (RR, 0.93; 95% CI 0.76–1.14). In the present study, we were not able to evaluate differences between gestational hypertension and preeclampsia due to the small sample size.

A meta-analysis including five cohort studies found no statistically significant association between prepregnancy physical activity and risk of preterm birth [17], which is in accordance with our findings.

To the best of our knowledge, no previous studies have explored the association between prepregnancy physical activity and risk of SGA offspring. However, several studies have examined the association between physical activity in pregnancy and risk of SGA. A meta-analysis of 4 randomized trials (754 women in exercise groups and 745 in control groups) found no evidence of an effect of exercise interventions in pregnancy on the risk of SGA offspring [32]. Likewise, a small cohort study from the USA including 291 women found no significant association between moderate or vigorous physical activity in the second and third trimester and risk for SGA [33]. In contrast, a cohort study conducted among women in Northern California reported an association between high levels of MVPA during the first trimester and an increased risk of SGA offspring, particularly among underweight and normal-weight women [34].

### Strengths and limitations

To the best of our knowledge, this is one of the first studies to assess the association between accelerometer-measured physical activity prior to pregnancy and risk of APOs.

The use of objectively measure physical activity is a major strength of our study. Most previous studies have relied on self-reported measures of physical activity, which are limited by the tendency for individuals to overestimate their physical activity levels, particularly among those who are less active. This overreporting and differential misclassification can lead to inaccuracies in assessing the true relationship between physical activity and APO [35]. Further strengths of this study include the population-based design and the linkage to high quality outcome data from a national birth registry. We were able to adjust our analyses for a broad range of confounders, particularly including those related to healthy lifestyle pattern, such as standardized measures of BMI, smoking, alcohol intake and self-reported general health. However, we did not have information on healthy diet and, as in all observational studies, we cannot rule out the possibility of residual confounding.

This study investigated the association of physical activity and the composite variable of multiple APOs, but the impact of physical activity may vary across different types of APOs. However, this approach provides a broader understanding of the potential influence of physical activity on APOs, highlighting the importance of including advice on physical activity into preconceptional counseling.

A limitation of our study is the relatively small sample size, which affect the precision of the results. However, despite this limitation, the study provides valuable insights into the association of objectively measured physical activity before pregnancy and risk of APOs that can inform former research. Study inclusion was related to healthy lifestyle pattern, which may have led to selection bias and limited the generalizability of the results. The proportion of APOs in our study population differed slightly from national average risks but was comparable to the proportions reported for Trøndelag county during the study period, except for a slightly higher proportion of women with preterm birth [36]. Finally, the findings from this study population may not be generalizable to broader, more diverse populations.

## Conclusions

Increased physical activity, including everyday activities, may reduce the risk of GDM and potentially the risk of other APOs. Encouraging women to increase their physical activity before pregnancy, particularly by incorporating daily activities that contribute to total activity levels, including light-intensity movement, could be an effective strategy for improving pregnancy health. However, larger studies are needed to confirm these findings and to further clarify the relationship between APOs and physical activity.

## Electronic supplementary material

Below is the link to the electronic supplementary material.

Supplementary Material 1

## Data Availability

The datasets generated and/or analysed during the current study are not publicly available due to restrictions imposed by the HUNT Research Centre (in accordance with the Norwegian Data Inspectorate) but are available from the corresponding author on reasonable request. Data are currently stored in the HUNT databank, and there are restrictions in place for the handling of HUNT data files. Data used from the HUNT Study in research projects will be made available on request to the HUNT Data Access Committee (kontakt@hunt.ntnu.no). The HUNT data access information (available here: http://www.ntnu.edu/hunt/data) describes in detail the policy regarding data availability.

## Abbreviations

APOs: Adverse pregnancy outcomes
BMI: Body mass index
CI: Confidence interval
GDM: Gestational diabetes mellitus
HUNT: Trøndelag Health study
HDP: Hypertensive disorders of pregnancy
MBRN: Medical Birth Registry of Norway
MET: Metabolic equivalents of task
MVPA: Moderate to vigorous physical activity
OR: Odds ratio
SGA: Small for gestational age
SD: Standard deviation
WHO: World Health Organization
