# Supplementary material for: Device measured physical activity before pregnancy and the risk of adverse pregnancy outcomes in the HUNT study: a population-based cohort study
Source: BMC Pregnancy Childbirth. 2025 Jun 7;25:661. doi: 10.1186/s12884-025-07779-7 (PMC12145615; doi:10.1186/s12884-025-07779-7)
Supplement: Supplementary file 1 — Supplementary Material 1 [file 12884_2025_7779_MOESM1_ESM.docx]

# **Supplementary material**

**Device measured physical activity before pregnancy and the risk of adverse pregnancy outcomes in the HUNT study: a prospective cohort study**

| Supplemental Table 1. Association of prepregnancy physical activity with the risk of adverse pregnancy outcomes among HUNT4 participants without self-reported moderate or severe impairment due to physical or mental health problems (n=663) | | | | | | | | | | | |
| --- | --- | --- | --- | --- | --- | --- | --- | --- | --- | --- | --- |
|  | | | | Number of cases | | Model 1^a^ | | Model 2^b^ | | Model 3^c^ | |
|  |  | | | | | OR | 95% CI | OR | 95% CI | OR | 95% CI |
| Any adverse pregnancy outcome | | | | | **138** |  |  |  |  |  |  |
|  | Total physical activity/day | |  | | |  |  |  |  |  |  |
|  |  | <33 percentile | 58 | | | Ref. |  | Ref. |  | Ref. |  |
|  |  | 33-66 percentile | 41 | | | 0.63 | (0.40-1.00) | 0.66 | (0.41-1.04) | 0.68 | (0.42-1.08) |
|  |  | >66 percentile | 39 | | | 0.57 | (0.36-0.91) | 0.63 | (0.39-1.01) | 0.66 | (0.41-1.07) |
|  | MVPA (MET min/week) | |  | | |  |  |  |  |  |  |
|  |  | <500 | 23 | | | Ref. |  | Ref. |  | Ref. |  |
|  |  | 500-1000 | 50 | | | 0.63 | (0.36-1.12) | 0.56 | (0.31-1.01) | 0.59 | (0.33-1.07) |
|  |  | >1000 | 65 | | | 0.71 | (0.41-1.23) | 0.58 | (0.33-1.03) | 0.63 | (0.35-1.15) |
| HDP | | | **36** | | |  |  |  |  |  |  |
|  | Total physical activity/day | |  | | |  |  |  |  |  |  |
|  |  | <33 percentile | 17 | | | Ref. |  | Ref. |  | Ref. |  |
|  |  | 33-66 percentile | 10 | | | 0.56 | (0.25-1.25) | 0.57 | (0.25-1.29) | 0.59 | (0.26-1.36) |
|  |  | >66 percentile | 9 | | | 0.48 | (0.21-1.12) | 0.53 | (0.23-1.24) | 0.57 | (0.24-1.38) |
|  | MVPA (MET min/week) | |  | | |  |  |  |  |  |  |
|  |  | <500 | 8 | | | Ref. |  | Ref. |  | Ref. |  |
|  |  | 500-1000 | 12 | | | 0.46 | (0.18-1.18) | 0.41 | (0.16-1.06) | 0.45 | (0.17-1.18) |
|  |  | >1000 | 16 | | | 0.53 | (0.22-1.28) | 0.43 | (0.17-1.08) | 0.50 | (0.19-1.29) |
| GDM | | | **24** | | |  |  |  |  |  |  |
|  | Total physical activity/day | |  | | |  |  |  |  |  |  |
|  |  | <33 percentile | 10 | | | Ref. |  | Ref. |  | Ref. |  |
|  |  | 33-66 percentile | 12 | | | 1.15 | (0.48-2.73) | 1.19 | (0.49-2.89) | 1.58 | (0.61-4.09) |
|  |  | >66 percentile | 2 | | | 0.16 | (0.03-0.73) | 0.16 | (0.03-0.74) | 0.18 | (0.04-0.90) |
|  | MVPA (MET min/week) | |  | | |  |  |  |  |  |  |
|  |  | <500 | 5 | | | Ref. |  | Ref. |  | Ref. |  |
|  |  | 500-1000 | 12 | | | 0.74 | (0.25-2.18) | 0.79 | (0.26-2.37) | 1.02 | (0.33-3.19) |
|  |  | >1000 | 7 | | | 0.34 | (0.10-1.10) | 0.37 | (0.11-1.25) | 0.55 | (0.15-1.96) |
| SGA | | | **53** | | |  |  |  |  |  |  |
|  | Total physical activity/day | |  | | |  |  |  |  |  |  |
|  |  | <33 percentile | 23 | | | Ref. |  | Ref. |  | Ref. |  |
|  |  | 33-66 percentile | 14 | | | 0.59 | (0.29-1.19) | 0.61 | (0.30-1.22) | 0.53 | (0.26-1.10) |
|  |  | >66 percentile | 16 | | | 0.70 | (0.36-1.38) | 0.77 | (0.39-1.52) | 0.65 | (0.32-1.31) |
|  | MVPA (MET min/week) | |  | | |  |  |  |  |  |  |
|  |  | <500 | 8 | | | Ref. |  | Ref. |  | Ref. |  |
|  |  | 500-1000 | 19 | | | 0.76 | (0.32-1.81) | 0.66 | (0.27-1.61) | 0.57 | (0.23-1.40) |
|  |  | >1000 | 26 | | | 0.90 | (0.39-2.07) | 0.72 | (0.31-1.71) | 0.61 | (0.25-1.50) |
| Preterm Birth | | | **42** | | |  |  |  |  |  |  |
|  | Total physical activity/day | |  | | |  |  |  |  |  |  |
|  |  | <33 percentile | 11 | | | Ref. |  | Ref. |  | Ref. |  |
|  |  | 33-66 percentile | 13 | | | 1.17 | (0.51-2.68) | 1.27 | (0.55-2.93) | 1.16 | (0.49-2.71) |
|  |  | >66 percentile | 18 | | | 1.59 | (0.72-3.47) | 1.89 | (0.86-4.18) | 1.80 | (0.79-4.09) |
|  | MVPA (MET min/week) | |  | | |  |  |  |  |  |  |
|  |  | <500 | 4 | | | Ref. |  | Ref. |  | Ref. |  |
|  |  | 500-1000 | 11 | | | 0.88 | (0.27-2.83) | 0.80 | (0.24-2.61) | 0.73 | (0.22-2.43) |
|  |  | >1000 | 27 | | | 1.91 | (0.65-5.63) | 1.65 | (0.55-4.96) | 1.45 | (0.47-4.49) |
| HDP, GDM and/or SGA | | | **101** | | |  |  |  |  |  |  |
|  | Total physical activity/day | |  | | |  |  |  |  |  |  |
|  |  | <33 percentile | 49 | | | Ref. |  | Ref. |  | Ref. |  |
|  |  | 33-66 percentile | 29 | | | 0.53 | (0.32-0.88) | 0.54 | (0.33-0.91) | 0.58 | (0.34-0.97) |
|  |  | >66 percentile | 23 | | | 0.40 | (0.23-0.69) | 0.44 | (0.25-0.77) | 0.47 | (0.27-0.83) |
|  | MVPA (MET min/week) | |  | | |  |  |  |  |  |  |
|  |  | <500 | 20 | | | Ref. |  | Ref. |  | Ref. |  |
|  |  | 500-1000 | 40 | | | 0.58 | (0.32-1.07) | 0.53 | (0.28-0.98) | 0.58 | (0.30-1.09) |
|  |  | >1000 | 41 | | | 0.51 | (0.28-0.94) | 0.43 | (0.23-0.81) | 0.50 | (0.26-0.96) |
| APO, adverse pregnancy outcome; BMI, body mass index; CI, confidence interval; GDM, gestational diabetes mellitus; HDP, hypertensive disorder of pregnancy; MET, metabolic equivalent of task; MVPA, moderate-vigorous physical activity; OR, odds ratio; SGA, small for gestational age.  Total physical activity/day percentiles: <33 = 0-89 min, 33-66 = 89-115 min, >66 = > 115 min.  a: adjusted for age. b: adjusted for age, living with partner/no partner, education and parity.  c: additionally adjusted for smoking, alcohol intake, BMI and self-reported general health. | | | | | | | | | | | |

| Supplemental Table 2. Logistic regression analysis. Association of preconception physical activity with the risk of adverse pregnancy outcomes among HUNT4 participants, Included unreliable measurements of PA. (n=735) | | | | | | | | | |
| --- | --- | --- | --- | --- | --- | --- | --- | --- | --- |
|  |  | | Number of cases | Model 1^a^ | | Model 2^b^ | | Model 3^c^ | |
|  | | |  | OR | 95% CI | OR | 95% CI | OR | 95% CI |
| Any adverse pregnancy outcome | | |  |  |  |  |  |  |  |
|  | Total physical activity/day | | **152** |  |  |  |  |  |  |
|  |  | <33 percentile | 65 | Ref. |  | Ref. |  | Ref. |  |
|  |  | 33-66 percentile | 46 | 0.74 | (0.48-1.14) | 0.78 | (0.50-1.20) | 0.80 | (0.52-1.25) |
|  |  | >66 percentile | 41 | 0.65 | (0.42-1.01) | 0.70 | (0.45-1.10) | 0.73 | (0.46-1.16) |
|  | MVPA (MET min/week) | |  |  |  |  |  |  |  |
|  |  | <500 | 27 | Ref. |  | Ref. |  | Ref. |  |
|  |  | 500-1000 | 56 | 0.69 | (0.41-1.16) | 0.64 | (0.38-1.10) | 0.67 | (0.39-1.15) |
|  |  | >1000 | 69 | 0.78 | (0.47-1.31) | 0.68 | (0.40-1.16) | 0.74 | (0.43-1.26) |
| HDP | | | **40** |  |  |  |  |  |  |
|  | Total physical activity/day | |  |  |  |  |  |  |  |
|  |  | <33 percentile | 19 | Ref. |  | Ref. |  | Ref. |  |
|  |  | 33-66 percentile | 12 | 0.69 | (0.33-1.45) | 0.71 | (0.34-1.51) | 0.75 | (0.35-1.63) |
|  |  | >66 percentile | 9 | 0.52 | (0.23-1.18) | 0.56 | (0.24-1.28) | 0.61 | (0.26-1.44) |
|  | MVPA (MET min/week) | |  |  |  |  |  |  |  |
|  |  | <500 | 9 | Ref. |  | Ref. |  | Ref. |  |
|  |  | 500-1000 | 14 | 0.54 | (0.22-1.29) | 0.49 | (0.20-1.19) | 0.54 | (0.22-1.32) |
|  |  | >1000 | 17 | 0.60 | (0.26-1.39) | 0.50 | (0.21-1.19) | 0.57 | (0.23-1.38) |
| GDM | | | **25** |  |  |  |  |  |  |
|  | Total physical activity/day | |  |  |  |  |  |  |  |
|  |  | <33 percentile | 10 | Ref. |  | Ref. |  | Ref. |  |
|  |  | 33-66 percentile | 13 | 1.42 | (0.61-3.32) | 1.47 | (0.62-3.49) | 1.79 | (0.71-4.50) |
|  |  | >66 percentile | 2 | 0.19 | (0.04-0.87) | 0.19 | (0.04-0.90) | 0.22 | (0.04-1.07) |
|  | MVPA (MET min/week) | |  |  |  |  |  |  |  |
|  |  | <500 | 5 | Ref. |  | Ref. |  | Ref. |  |
|  |  | 500-1000 | 13 | 0.94 | (0.33-2.72) | 1.04 | (0.35-3.07) | 1.23 | (0.40-3.73) |
|  |  | >1000 | 7 | 0.42 | (0.13-1.36) | 0.48 | (0.14-1.60) | 0.62 | (0.18-2.26) |
| SGA | | | **59** |  |  |  |  |  |  |
|  | Total physical activity/day | |  |  |  |  |  |  |  |
|  |  | <33 percentile | 26 | Ref. |  | Ref. |  | Ref. |  |
|  |  | 33-66 percentile | 15 | 0.64 | (0.33-1.24) | 0.65 | (0.33-1.27) | 0.59 | (0.30-1.17) |
|  |  | >66 percentile | 18 | 0.81 | (0.43-1.54) | 0.86 | (0.46-1.64) | 0.73 | (0.38-1.41) |
|  | MVPA (MET min/week) | |  |  |  |  |  |  |  |
|  |  | <500 | 9 | Ref. |  | Ref. |  | Ref. |  |
|  |  | 500-1000 | 23 | 0.91 | (0.41-2.05) | 0.85 | (0.37-1.91) | 0.74 | (0.32-1.70) |
|  |  | >1000 | 27 | 0.99 | (0.45-2.18) | 0.85 | (0.38-1.90) | 0.73 | (0.32-1.67) |
| Preterm Birth | | | **49** |  |  |  |  |  |  |
|  | Total physical activity/day | |  |  |  |  |  |  |  |
|  |  | <33 percentile | 15 | Ref. |  | Ref. |  | Ref. |  |
|  |  | 33-66 percentile | 15 | 1.12 | (0.54-2.35) | 1.20 | (0.57-2.52) | 1.16 | (0.54-2.46) |
|  |  | >66 percentile | 19 | 1.45 | (0.71-2.94) | 1.62 | (0.79-3.31) | 1.66 | (0.79-3.45) |
|  | MVPA (MET min/week) | |  |  |  |  |  |  |  |
|  |  | <500 | 8 | Ref. |  | Ref. |  | Ref. |  |
|  |  | 500-1000 | 11 | 0.47 | (0.19-1.21) | 0.46 | (0.18-1.19) | 0.44 | (0.17-1.15) |
|  |  | >1000 | 30 | 1.24 | (0.55-2.80) | 1.16 | (0.51-2.68) | 1.15 | (0.49-2.70) |
| APOs except preterm birth | | | **109** |  |  |  |  |  |  |
|  | Total physical activity/day | |  |  |  |  |  |  |  |
|  |  | <33 percentile | 53 | Ref. |  | Ref. |  | Ref. |  |
|  |  | 33-66 percentile | 32 | 0.63 | (0.39-1.03) | 0.66 | (0.40-1.07) | 0.70 | (0.43-1.15) |
|  |  | >66 percentile | 24 | 0.47 | (0.28-0.79) | 0.51 | (0.30-0.86) | 0.53 | (0.31-0.92) |
|  | MVPA (MET min/week) | |  |  |  |  |  |  |  |
|  |  | <500 | 21 | Ref. |  | Ref. |  | Ref. |  |
|  |  | 500-1000 | 46 | 0.73 | (0.41-1.29) | 0.69 | (0.38-1.24) | 0.74 | (0.40-1.34) |
|  |  | >1000 | 42 | 0.61 | (0.34-1.10) | 0.54 | (0.29-0.98) | 0.59 | (0.32-1.10) |
| APO, adverse pregnancy outcome; HDP, hypertensive disorder of pregnancy; GDM, gestational diabetes mellitus; SGA, small for gestational age; OR, odds ratio; CI, confidence interval; MVPA, moderate-vigorous physical activity; MET, metabolic equivalent of task. Total physical activity/day percentiles: <33 = 0-89 min, 33-66 = 89-115 min, >66 = > 115 min.  a: adjusted for age. b: adjusted for age, living with partner/no partner, education and parity.  c: additionally adjusted for smoking, alcohol intake, BMI and self-reported general health. | | | | | | | | | |

| Supplemental Table 3. Descriptive characteristics of HUNT4 participants with childbirth after HUNT4 according to inclusion status | | | | |
| --- | --- | --- | --- | --- |
| Characteristics | | **All women (n=2051)** | **Excluded from the analysis (n=1351)** | **Included in the analysis**  **(n=700)** |
| Age, years, mean (SD) | | 28.3 (4.6) | 28.3 (4.7) | 28.3 (4.4) |
| Living with partner | | 1650 (80.5) | 1103 (81.6) | 547 (78.1) |
| Education | |  |  |  |
|  | Professional certificate, high school or less | 784 (38.2) | 565 (41.8) | 219 (31.3) |
|  | Greater than high school | 1249 (60.9) | 768 (56.9) | 481 (68.7) |
|  | Missing | 18 (0.9) | 18 (1.3) | 0 (0.0) |
| Parity | |  |  |  |
|  | Nulliparous | 981 (47.8) | 622 (46.0) | 359 (51.3) |
|  | Parous | 1070 (52.2) | 729 (54.0) | 341 (48.7) |
| General health | |  |  |  |
|  | Poor/fair | 230 (11.2) | 159 (11.8) | 71 (10.1) |
|  | Good/excellent | 1803 (87.9) | 1174 (86.9) | 629 (89.9) |
|  | Missing | 18 (0.9) | 18 (1.3) | 0 (0.0) |
| Smoking status | |  |  |  |
|  | Never smoked | 1238 (60.4) | 787 (58.3) | 451 (64.4) |
|  | Ever smoked | 794 (38.7) | 545 (40.3) | 249 (35.6) |
|  | Missing | 19 (0.9) | 19 (1.4) | 0 (0.0) |
| Alcohol intake | |  |  |  |
|  | No alcohol consumption | 505 (24.6) | 340 (25.2) | 165 (23.6) |
|  | Any alcohol consumption | 1308 (63.8) | 773 (57.2) | 535 (76.4) |
|  | Missing | 238 (11.6) | 238 (17.6) | 0 (0.0) |
| BMI categories, kg/m^2^ | |  |  |  |
|  | Underweight/normal weight | 1039 (50.7) | 630 (46.6) | 409 (58.4) |
|  | Overweight | 565 (27.6) | 384 (28.4) | 181 (25.9) |
|  | Obese | 355 (17.3) | 245 (18.1) | 110 (15.7) |
|  | Missing | 92 (4.5) | 92 (6.8) | 0 (0.0) |
| Self-reported physical activity | |  |  |  |
|  | < 1 time/week | 332 (16.2) | 235 (17.4) | 97 (13.9) |
|  | 1 time/week | 391 (19.1) | 256 (19.0) | 135 (19.3) |
|  | 2-3 times /week | 906 (44.2) | 600 (44.4) | 306 (43.7) |
|  | > 3 times/week | 391 (19.1) | 232 (17.2) | 159 (22.7) |
|  | Missing | 31 (1.5) | 28 (2.1) | 3 (0.4) |
| Self-reported PA, combined variable | |  |  |  |
|  | Inactive | 679 (33.1) | 452 (33.5) | 227 (32.4) |
|  | Active | 992 (48.4) | 625 (46.3) | 367 (52.4) |
|  | Missing | 380 (18.5) | 274 (20.3) | 106 (15.1) |
| Values are presented as n (%) for categorical variables and mean (SD) for continuous variables. APO, adverse pregnancy outcome; GDM, gestational diabetes mellitus; HDP, hypertensive disorders of pregnancy; SD, standard deviation; SGA, small for gestational age. | | | | |
